# High-dose ruxolitinib (25 mg twice daily) in myelofibrosis: feasibility, safety, and long-term treatment exposure in a real-world cohort

**DOI:** 10.1007/s00277-026-07020-1

**Published:** 2026-05-04

**Authors:** Francesco Mendicino, Giulio Caridà, Antonella Bruzzese, Enrica Antonia Martino, Santino Caserta, Nicola Amodio, Eugenio Lucia, Virginia Olivito, Caterina Labanca, Maria Eugenia Alvaro, Filippo Luciani, Fortunato Morabito, Ernesto Vigna, Massimo Gentile

**Affiliations:** 1 Department of Onco-Haematology, Haematology Unit, Haematology Unit, AO of Cosenza, Cosenza, 87100 Italy; 2 Department of Experimental and Clinical Medicine, University of Catanzaro, Catanzaro, Italy; 3 Azienda Sanitaria Provinciale (ASP) of Cosenza, Cosenza, Italy; 4 Associazione Italiana Contro Le Leucemie-Linfomi E Mieloma” Section of Cosenza, Cosenza, Italy; 5 Department of Pharmacy, Health and Nutritional Science, University of Calabria, Rende, Italy

**Keywords:** Myelofibrosis, Ruxolitinib, Dose intensity, Real-world evidence, JAK inhibitors, High-dose therapy

## Abstract

**Background:**

Ruxolitinib is standard first-line therapy for symptomatic myelofibrosis (MF). In real-world practice, dose reductions are common, and the impact of maintaining higher dose intensity over time remains incompletely characterized—particularly for patients escalated to 25 mg twice daily (50 mg/day; full dose).

**Methods:**

We conducted a single-center retrospective cohort study of consecutive MF patients (2015–2025) who received 25 mg BID at least once during their treatment course. Data were extracted from a structured institutional database linking baseline (DEMOGRAPHY) and visit-level longitudinal data (HISTORY), including dosing, supportive care, infections, transfusions, and spleen measurements. Dose-intensified exposure was defined as 40 mg/day or higher and was used to anchor t0 (first visit at 40 mg/day or higher), while full-dose exposure specifically referred to the approved maximum of 50 mg/day (25 mg twice daily). FRAC_EQ50 quantified sustained full-dose exposure as the fraction of visits at 50 mg/day. Analyses were descriptive.

**Results:**

Twenty-four patients were included, with a median follow-up of 60.9 months from ruxolitinib initiation. Based on FRAC_EQ50, 11/24 patients were classified as high-intensity (FRAC_EQ50 0.50–1.00), 6/24 as intermediate (0.10–0.49), and 7/24 as minimal (below 0.10). For spleen-response analyses, baseline was defined as the first ultrasound spleen length recorded after t0 and was available in 22/24 patients; spleen response was evaluable in 21/24 (one patient lacked a follow-up ultrasound with spleen length in cm; two had no evaluable post-baseline spleen assessments). Spleen length decreased in 17/21 evaluable patients (81.0%), while hematologic toxicities and supportive-care needs remained manageable across intensity groups. Four deaths occurred during follow-up, including one fatal COVID-19 case temporally associated with abrupt ruxolitinib discontinuation during ICU intubation.

**Conclusions:**

In selected MF patients, escalation to 25 mg BID with sustained full-dose intensity appears feasible in routine practice, with durable treatment exposure and manageable safety. These real-world data support further multicenter efforts to better characterize longitudinal dose exposure and its potential clinical implications, without implying causal relationships.

**Supplementary Information:**

The online version contains supplementary material available at 10.1007/s00277-026-07020-1.

## Introduction

Myelofibrosis (MF) is a clonal myeloproliferative neoplasm characterized by bone marrow fibrosis, progressive splenomegaly, constitutional symptoms, cytopenias, and reduced survival. Its biological hallmark is a pro-inflammatory, cytokine-driven microenvironment sustained by constitutive activation of the JAK–STAT signaling pathway [1]. Clinical heterogeneity in MF is further shaped by the underlying genomic landscape, as reflected in contemporary molecular classifications and prognostic models [2]. Over the past decade, ruxolitinib has become the standard first-line therapy for symptomatic MF, including international guideline-aligned recommendations and contemporary expert updates on MF management [3, 4].

The pivotal COMFORT trials established the efficacy of ruxolitinib in reducing splenomegaly and disease-related symptoms, with COMFORT-I demonstrating superiority over placebo [5] and COMFORT-II confirming benefit compared with best available therapy [6]. Long-term follow-up of COMFORT-II further showed durable clinical responses and survival advantages over extended observation periods [7]. These findings have been corroborated by large real-world cohorts, including national and international datasets, which confirmed the effectiveness and safety of ruxolitinib across heterogeneous clinical settings [8, 9]. In addition, data from the expanded-access JUMP study highlighted how baseline clinical and laboratory characteristics influence treatment response, underscoring the complexity of response prediction in MF [10].

Despite these advances, the optimal dosing strategy of ruxolitinib remains an area of active investigation. Growing evidence suggests that adequate dose intensity is a key determinant of treatment benefit. Several real-world analyses have shown that higher cumulative dose exposure is associated with improved survival and more durable responses [11, 12], whereas initiation at reduced doses has been linked to inferior outcomes in large contemporary registries [13]. Post-COVID analyses from national AIFA databases have further documented a trend toward more conservative starting doses, although the impact of such strategies on long-term outcomes remains debated after adjustment for confounding factors [14].

While the consequences of under-dosing have been increasingly characterized, the feasibility and long-term outcomes of dose-intensified ruxolitinib—particularly sustained exposure to the maximum approved dose of 25 mg twice daily (50 mg/day)—remain far less well documented in routine clinical practice. Prospective studies such as EXPAND have demonstrated that dose escalation can be feasible and safe even in selected patients with baseline thrombocytopenia [15]. However, real-world data describing long-term feasibility, safety, treatment trajectories, and reasons for discontinuation at high dose intensity are scarce, especially beyond the controlled environment of clinical trials.

To address this gap, we conducted a detailed real-world analysis of consecutive MF patients treated at our institution with dose-intensified ruxolitinib, including sustained exposure to the full approved dose (25 mg twice daily). The objectives of this study were to characterize longitudinal treatment exposure, spleen response, hematologic and non-hematologic safety, infection risk, second malignancies, and clinical outcomes, with particular emphasis on dose intensity and long-term feasibility in routine practice.

## Materials and methods

### Study design and data sources

We conducted a single-center, retrospective cohort study including consecutive patients with myelofibrosis (primary MF, post–polycythemia vera MF, or post–essential thrombocythemia MF) treated with ruxolitinib between 2015 and 2025, with planned escalation to dose-intensified regimens including the maximum approved dose of 25 mg twice daily. Clinical data were extracted from a structured institutional database composed of two linked worksheets:

- **DEMOGRAPHY**, containing baseline characteristics, disease features, prior therapies, recalculated DIPSS risk category, and outcomes;
- **HISTORY**, containing visit-level information including laboratory values, spleen measurements, transfusions, infections, ESA use, ruxolitinib dosing, and timing of switching to other JAK inhibitors.

### Eligibility and exposure definitions

Eligible patients were those who received ruxolitinib as part of routine clinical care and who were prescribed 25 mg twice daily at least once during their treatment course (dose intensification). Dose-intensified exposure was defined as 40 mg/day or higher (i.e., 20 mg twice daily or higher) and was used to anchor t0, defined as the first visit at 40 mg/day or higher. Full-dose exposure referred specifically to 50 mg/day (25 mg twice daily), the maximum approved daily dose. In this cohort, “full-dose” refers to the approved maximum dose (50 mg/day), whereas 40 mg/day was used only to define dose-intensified exposure and anchor t0. Full-dose intensity metric (FRAC_EQ50). The label “EQ50” indicates that the daily dose equals 50 mg/day. To quantify sustained exposure to the full approved dose, we calculated FRAC_EQ50 for each patient as the fraction of evaluable follow-up visits recorded at exactly 50 mg/day (25 mg twice daily): (number of visits with daily dose = 50 mg/day) / (total number of visits with non-missing dose information). Visits with missing dose data were excluded from the denominator. Daily dose (mg/day) was derived from the visit-level variable TOTAL_DOSAGE_RUXOLITINIB recorded over 28 days in the HISTORY worksheet using: dose (mg/day) = TOTAL_DOSAGE_RUXOLITINIB/28. Patients were stratified into three full-dose intensity categories: high (FRAC_EQ50 0.50–1.00), intermediate (0.10–0.49), and minimal (below 0.10).

### Variables of interest

Baseline variables included age, sex, MF subtype, driver mutation status (JAK2/CALR/MPL), DIPSS risk category, spleen size, blood counts, and comorbidities. Visit-level variables included ruxolitinib dose, ESA use, transfusions, infections, and longitudinal spleen measurements.

### Safety

Hematologic toxicity was assessed using CTCAE v5.0. Non-hematologic adverse events, infections, and second malignancies were captured from the HISTORY worksheet and verified by manual chart review. Non-melanoma skin cancer (NMSC) was defined as histologically confirmed basal cell carcinoma or squamous cell carcinoma.

### Spleen assessment

Baseline spleen size was obtained from the DEMOGRAPHY worksheet when available. Longitudinal spleen response was evaluated using ultrasound-based spleen length (cm) recorded in the HISTORY worksheet and summarized as percentage change from baseline, using the best (minimum) post-baseline spleen measurement when serial assessments were available. Patients without an evaluable post-baseline spleen measurement expressed in centimeters were excluded from spleen response analyses.

### Statistical analysis

All analyses were descriptive. Continuous variables are reported as median (IQR), and categorical variables as n (%). Time to treatment discontinuation or switch was estimated using standard Kaplan–Meier methods. Given the retrospective design and the absence of time-dependent modeling of dose exposure, these analyses should be interpreted with caution. No inferential testing was performed due to sample size and the exploratory nature of the study. Formal time-to-event endpoints (e.g., progression-free survival or duration of response) were not calculated due to the retrospective design, limited sample size, and absence of standardized response-assessment schedules; therefore, treatment exposure and discontinuation were described descriptively, and time to discontinuation/switch was summarized as an exploratory endpoint (Supplementary Figure S3).

## Results

### Patient characteristics and follow-up

A total of 24 consecutive patients with myelofibrosis were included in the analysis. Median follow-up from ruxolitinib initiation was 60.9 months (IQR 29.0–83.9). Four patients (4/24, 16.7%) died during follow-up (CS05, CS10, CS11, and CS19). Causes of death included severe COVID-19–related complications in one patient (CS05) and disease- or comorbidity-related causes in the remaining cases.

Baseline demographic, clinical, and hematologic characteristics are summarized in Table 1. For spleen-response analyses, baseline was defined as the first ultrasound spleen length recorded after t0 and was available for 22 of 24 patients, with a median value of 14.0 cm (IQR 11.5–17.0).

**Table 1:** Baseline demographic and clinical characteristics of the study cohort (*N* = 24) Continuous variables are reported as median (IQR); categorical variables as *n* (%)

Characteristic	Value
Age, years	69 (61–76)
Sex	
Male	16 (66.7%)
Female	8 (33.3%)
Myelofibrosis subtype	
Primary MF	14 (58.3%)
Post-PV MF	7 (29.2%)
Post-ET MF	3 (12.5%)
Driver mutation	
JAK2 V617F	19 (79.2%)
CALR	3 (12.5%)
MPL	1 (4.2%)
Triple-negative	1 (4.2%)
DIPSS risk category (baseline)	
Low/Intermediate-1	9 (37.5%)
Intermediate-2/High	15 (62.5%)
Hemoglobin, g/dL	13.0 (11.4–13.8)
Platelet count, × 10⁹/L	474.5 (331.5–772.3)
Baseline spleen length, cm*	14.0 (11.5–17.0)
Transfusion-dependent at baseline	3 (12.5%)
ESA use at baseline	9 (37.5%)

### Treatment exposure and dose-intensity trajectories

All patients received ruxolitinib as part of routine clinical care with escalation to dose-intensified regimens, including exposure to the maximum approved dose of 25 mg twice daily. Based on longitudinal treatment exposure quantified by FRAC_EQ50, patients were categorized into three dose-intensity groups according to the proportion of visits at full dose (50 mg/day): high-intensity, intermediate-intensity, and minimal-intensity exposure.

**Fig. 1:**
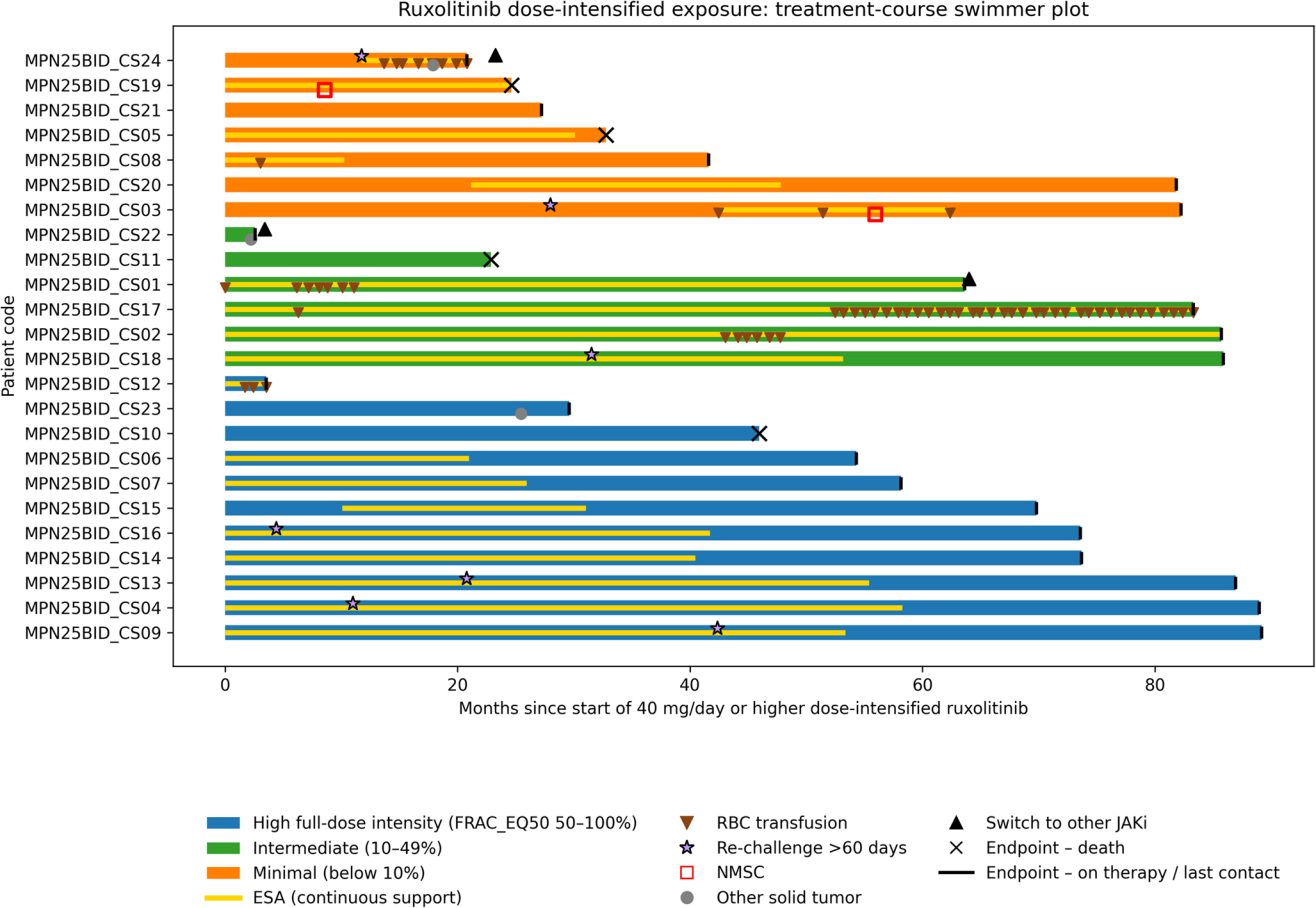
Treatment-course swimmer plot Swimmer plot of individual ruxolitinib treatment-course trajectories from the start of dose-intensified exposure (t0, first visit with at least 40 mg/day). Each horizontal bar represents the time on ruxolitinib after t0 and is color-coded by full-dose intensity category based on FRAC_EQ50 (proportion of follow-up visits at exactly 50 mg/day): high full-dose intensity (FRAC_EQ50 0.50–1.00), intermediate (0.10–0.49), and minimal (below 0.10). The internal yellow strip indicates continuous ESA support. Inverted brown triangles denote RBC transfusion events. Purple stars indicate ruxolitinib re-challenge after a treatment gap >60 days. Black upward triangles indicate switch to another JAK inhibitor. Red open squares indicate non-melanoma skin cancer (NMSC), and grey circles indicate other solid tumors. Black “X” indicates death; black vertical tick indicates patients still on therapy/last contact

Overall, 11 of 24 patients (45.8%) were classified as high-intensity, 6 of 24 (25.0%) as intermediate-intensity, and 7 of 24 (29.2%) as minimal-intensity. Individual dose trajectories over time are illustrated in Fig. 1, highlighting sustained full-dose exposure in a substantial proportion of patients. Visit-level daily dose trajectories stratified by FRAC_EQ50 categories are shown in Supplementary Figure S1, and the distribution of FRAC_EQ50 values across patients is summarized in Supplementary Figure S2. Time to ruxolitinib discontinuation or switch, measured from t0, is explored using Kaplan–Meier estimates in Supplementary Figure S3.

### Spleen response

Spleen response analyses were performed in 21 of 24 patients. Three patients were excluded due to lack of evaluable post-baseline spleen assessments: in one patient (CS03), baseline spleen size was available but no follow-up ultrasound with spleen measurements expressed in centimeters was documented; in two additional patients (CS10 and CS11), no evaluable post-baseline spleen measurements were available.

Among evaluable patients, median baseline spleen length was 14.0 cm (IQR 11.5–17.0). The median best percentage change from baseline was −16.5% (IQR − 21.5% to −9.1%). A reduction in spleen length was observed in 17 of 21 patients (81.0%). A spleen length reduction of 35% or greater (SVR35-like response) was documented in 1 of 21 patients (4.8%) when sufficient serial measurements were available.

**Fig. 2:**
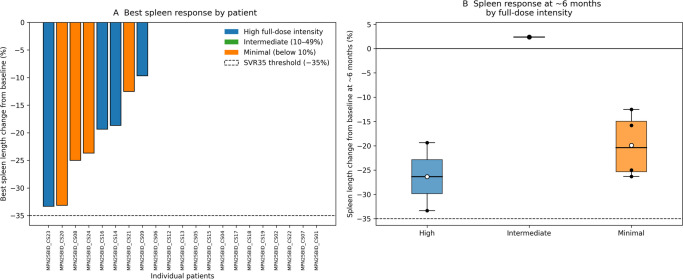
Spleen response from the start of dose-intensified exposure **A** Waterfall plot showing the maximum percentage change in spleen length from baseline for each patient (baseline defined as the first available spleen measurement after t0). The dashed line indicates the SVR35 threshold (− 35%). Bars are color-coded by full-dose intensity category based on FRAC_EQ50: High (0.50–1.00), Intermediate (0.10–0.49), and Minimal (below 0.10). **B** Boxplot of spleen length percentage change from baseline at ~6 months, defined as the measurement closest to 6 months within a 3–9 month window after the baseline assessment. Individual data points are overlaid; the mean is displayed as a white marker

When stratified by dose-intensity, spleen reduction occurred in 7 of 10 patients (70.0%) in the high-intensity group, 5 of 5 patients (100%) in the intermediate-intensity group, and 5 of 6 patients (83.3%) in the minimal-intensity group (Fig. 2).

### Hematologic toxicity and supportive care

Median hemoglobin at baseline was 13.0 g/dL (IQR 11.4–13.8), with a median nadir hemoglobin of 9.9 g/dL (IQR 8.6–11.8) during treatment. Median platelet count decreased from 474.5 × 10⁹/L (IQR 331.5–772.3) at baseline to a nadir of 170.0 × 10⁹/L (IQR 38.2–312.3).

**Fig. 3:**
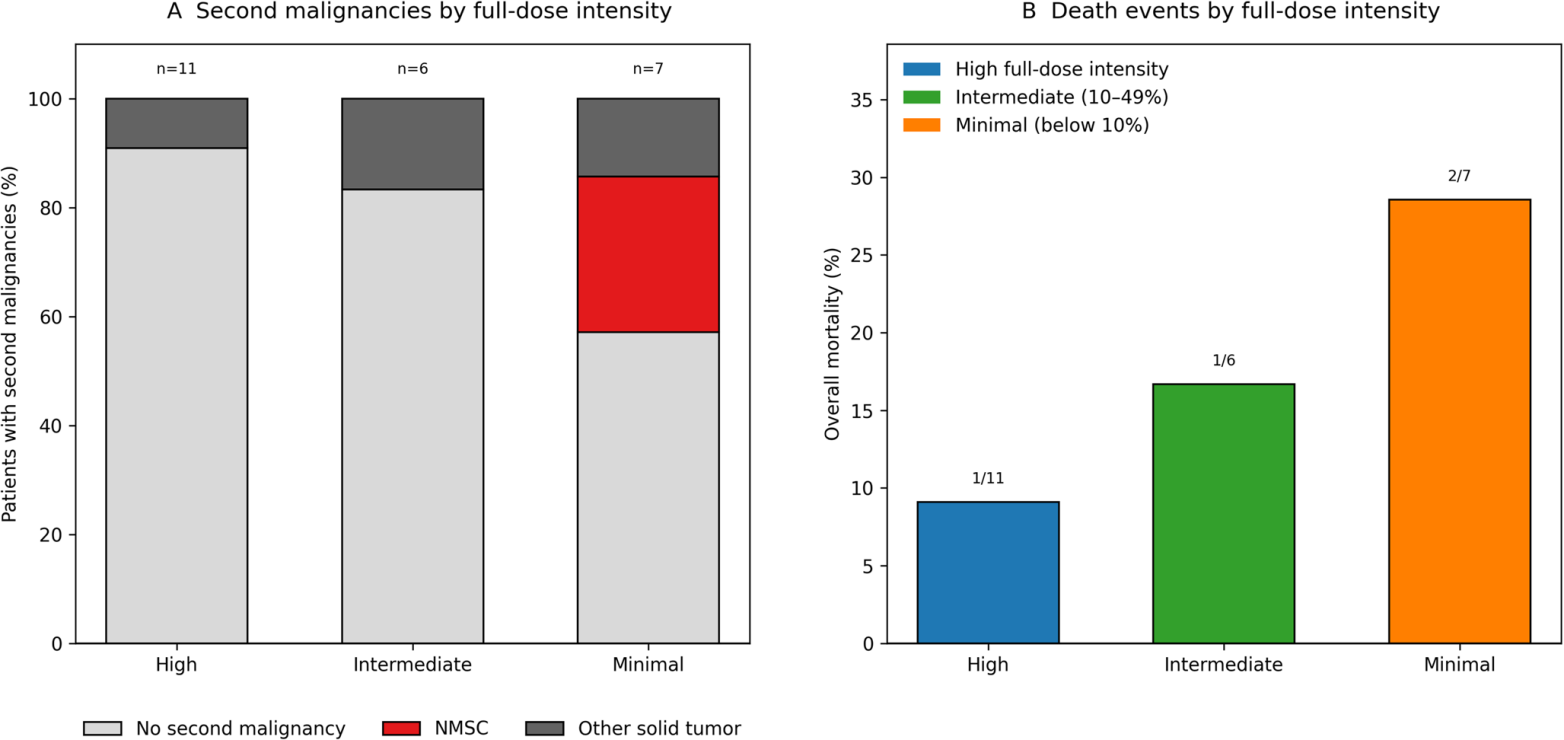
Second malignancies and mortality by full-dose intensity **A** Distribution of second malignancies by full-dose intensity category (FRAC_EQ50). Stacked bars display the percentage of patients with no second malignancy, NMSC, or other solid tumor, with the total number of patients in each category reported above bars. **B** Overall mortality by full-dose intensity category; values above bars indicate deaths/total in each group

Red blood cell transfusions were required in 3 of 24 patients (12.5%), while erythropoiesis-stimulating agents were administered in 14 of 24 patients (58.3%) during follow-up. Hematologic toxicities were manageable across dose-intensity groups and did not lead to systematic treatment discontinuation. Second malignancies and mortality according to dose intensity are summarized in Fig. 3.

### Safety, deaths, and second malignancies

Four patients died during follow-up. Second malignancies were observed in three patients, including one non-melanoma skin cancer and two solid tumors (cervical and colorectal carcinoma).

One patient (CS05) died from severe COVID-19–related complications requiring intensive care unit admission, following prolonged ruxolitinib exposure and abrupt treatment discontinuation during intubation. One patient (CS19) died from advanced pulmonary malignancy with metastatic disease and cardiorespiratory failure, on a background of long-standing cardiovascular comorbidities and prior non-melanoma skin cancers. The remaining deaths occurred in frail patients with advanced myelofibrosis and multiple comorbidities, following dose reductions, treatment discontinuation, or switching to alternative JAK inhibitors.

Overall, no clear association between sustained exposure to dose-intensified or full-dose ruxolitinib and mortality was observed.

## Discussion

Over the past decade, the therapeutic landscape of myelofibrosis (MF) has been profoundly shaped by the introduction of ruxolitinib. Its efficacy in reducing splenomegaly and symptom burden was first established in the pivotal COMFORT-I and COMFORT-II trials [5, 6], with long-term follow-up demonstrating durable clinical benefit and an association with improved overall survival [7]. Ten years after its approval, ruxolitinib remains the backbone of MF therapy, as highlighted by recent comprehensive reviews of its long-term efficacy and clinical impact [16]. These findings have been consistently confirmed by large real-world cohorts, including Italian and international datasets, which validated the safety and effectiveness of ruxolitinib across heterogeneous clinical settings [8, 9].

Despite this consolidated role, the optimal dosing strategy of ruxolitinib remains incompletely defined, and increasing attention has been directed toward the clinical relevance of dose intensity. Several real-world analyses have suggested that higher cumulative dose exposure may translate into improved disease control and survival [11, 12]. Conversely, data from large registries, including the AIFA database, have shown that initiation at reduced doses may be associated with inferior outcomes, even after adjustment for clinical confounders [13]. During the COVID-19 era, a complementary Annals of Hematology analysis documented a shift toward more conservative starting doses, although long-term persistence and survival appeared preserved after accounting for baseline risk profiles [14]. Together, these findings underscore the complex interplay between disease severity, patient selection, and dosing strategy, supporting the hypothesis that adequate dose intensity may be a clinically relevant determinant of long-term benefit.

In this context, our study provides focused real-world evidence on dose-intensified ruxolitinib, including sustained exposure to the maximum approved dose of 25 mg twice daily, a treatment approach that remains poorly characterized outside clinical trials. We show that, in a carefully selected patient population, prolonged exposure to full-dose ruxolitinib was feasible and associated with durable spleen reduction and long treatment continuity, without a disproportionate increase in hematologic or non-hematologic toxicity. Importantly, the present analysis was not designed to demonstrate superiority in efficacy outcomes, and any apparent differences in spleen response across dose-intensity categories should therefore be interpreted cautiously given the limited sample size and descriptive nature of the study. These findings are consistent with observations from dose-escalation studies such as EXPAND [15] and align with safety profiles reported in standard-dose real-world cohorts [8, 9], suggesting that higher dose intensity per se does not necessarily translate into excess toxicity when managed in experienced centers. Notably, higher full-dose intensity was not associated with clearly higher spleen response rates in this small cohort, underscoring the exploratory and feasibility-oriented nature of the study.

The incidence of second malignancies observed in our cohort was low and consistent with previously reported background rates in ruxolitinib-treated MF populations [11, 17]. Specifically, the occurrence of one non-melanoma skin cancer and two solid tumors did not suggest a dose-dependent oncologic signal, reinforcing the notion that sustained full-dose exposure does not inherently increase malignancy risk. Similarly, the profile of infectious complications was consistent with published real-world reports on ruxolitinib-treated patients [18], indicating that dose-intensified ruxolitinib can be administered safely with appropriate monitoring and supportive care. A particularly relevant observation concerns COVID-19–related outcomes. Early during the pandemic, abrupt ruxolitinib discontinuation was associated with cytokine rebound and high mortality in patients with myeloproliferative neoplasms [19], with subsequent analyses showing improved outcomes once unnecessary withdrawal was avoided [20]. Mechanistic and clinical reports further described exacerbation of hyperinflammatory states following sudden JAK inhibition interruption [21]. In our cohort, this phenomenon was exemplified by one fatal case occurring after abrupt ruxolitinib cessation during intensive care unit admission, reinforcing the critical importance of avoiding unplanned treatment interruption whenever feasible, especially in inflammatory or infectious contexts.

Beyond individual clinical events, the strength of our study lies in the granularity of longitudinal, visit-level data, allowing reconstruction of real-time dose trajectories, supportive therapies, and treatment context over extended follow-up. Such transparent and harmonized operational definitions are particularly relevant in real-world settings, where spleen response assessment and definitions of treatment failure are not uniformly applied across centers, thereby limiting cross-study comparability [22]. This level of detail provides a nuanced view of how ruxolitinib dose intensity is managed in everyday practice, a perspective that is largely lacking in the existing literature.

Several limitations should be acknowledged, including the modest sample size inherent to the strict inclusion of patients exposed to 25 mg twice daily, the retrospective single-center design, and the absence of a formal comparator cohort treated exclusively at standard doses or with alternative JAK inhibitors. In particular, patients escalated to and maintained on full-dose ruxolitinib represent a clinically selected subgroup, potentially enriched for individuals with better baseline hematologic reserve and treatment tolerance. Nevertheless, the internal consistency of treatment patterns, safety signals, and exposure–response relationships supports the robustness of our observations. In addition, the FRAC_EQ50 metric, while providing a practical summary of sustained full-dose exposure, has inherent limitations that should be acknowledged. Visit frequency was not standardized and was physician-driven, potentially introducing variability across patients. Although visits with missing dose data were excluded from the denominator, this may still result in information bias. Furthermore, the cut-offs used to define full-dose intensity categories were operationally defined within this dataset and should be considered arbitrary in the absence of external validation. Therefore, FRAC_EQ50 should be interpreted as an exploratory metric requiring validation in larger, prospective cohorts.

In summary, our study provides detailed real-world evidence supporting the feasibility, safety, and long-term treatment continuity of dose-intensified ruxolitinib, including sustained exposure to the full approved dose, in selected patients with MF. These findings integrate into a growing body of literature suggesting that adequate dose intensity may be a clinically relevant component of treatment strategy, and that, in appropriately selected patients, sustained JAK inhibition may be feasible, although no causal inference regarding clinical benefit or safety can be drawn in this setting. Accordingly, these findings should not be extrapolated indiscriminately to all patients with myelofibrosis, but rather viewed as representative of outcomes achievable in carefully selected patients managed in experienced centers. Taken together, our data highlight how detailed longitudinal treatment mapping can inform daily practice, refine therapeutic strategies, and guide future prospective studies aimed at maximizing the long-term benefits of JAK inhibition in myelofibrosis.

## Supplementary Information

Below is the link to the electronic supplementary material.

Supplementary file1 (DOCX 393 KB)

Supplementary file2 (DOCX 17 KB)

## Data Availability

Available upon reasonable request.
