# Supplementary material for: High-dose ruxolitinib (25 mg twice daily) in myelofibrosis: feasibility, safety, and long-term treatment exposure in a real-world cohort
Source: Ann Hematol. 2026 May 4;105(5):284. doi: 10.1007/s00277-026-07020-1 (PMC13139260; doi:10.1007/s00277-026-07020-1)
Supplement: Supplementary file 1 — Supplementary file1 (DOCX 393 KB) [file 277_2026_7020_MOESM1_ESM.docx]

**
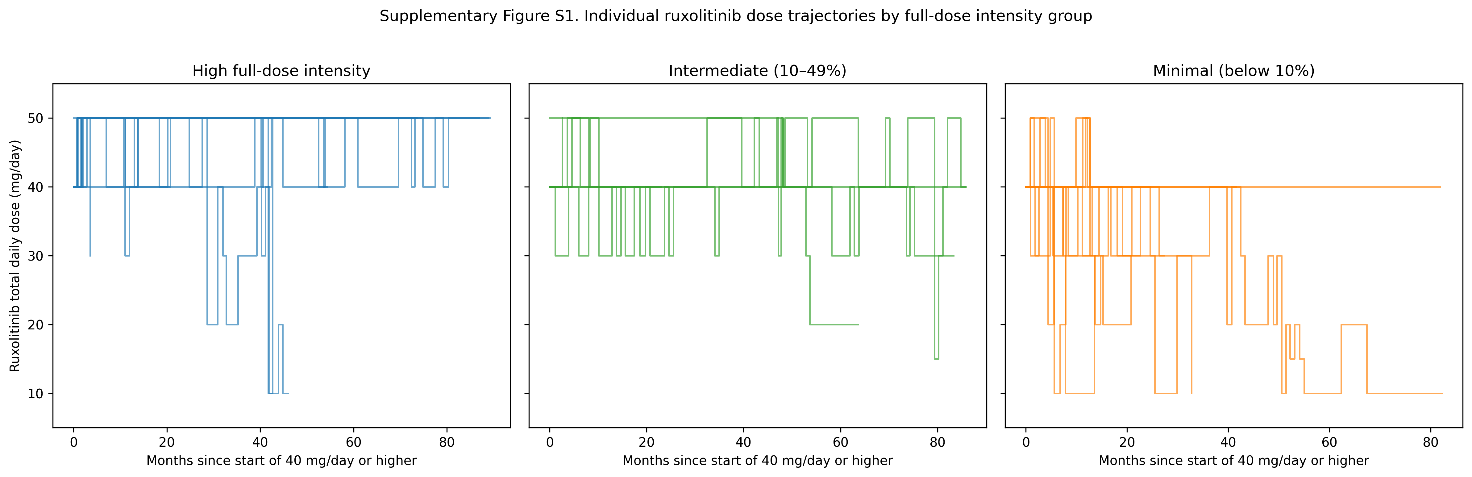
**

**Supplementary Figure S1.**

**Individual ruxolitinib dose trajectories**

Individual longitudinal ruxolitinib daily-dose trajectories from t0 (first visit at 40 mg/day or higher), stratified by full-dose intensity category based on FRAC_EQ50: High (FRAC_EQ50 0.50–1.00), Intermediate (0.10–0.49), and Minimal (<0.10). Curves represent visit-level dosing over time; category colors match the main figures.


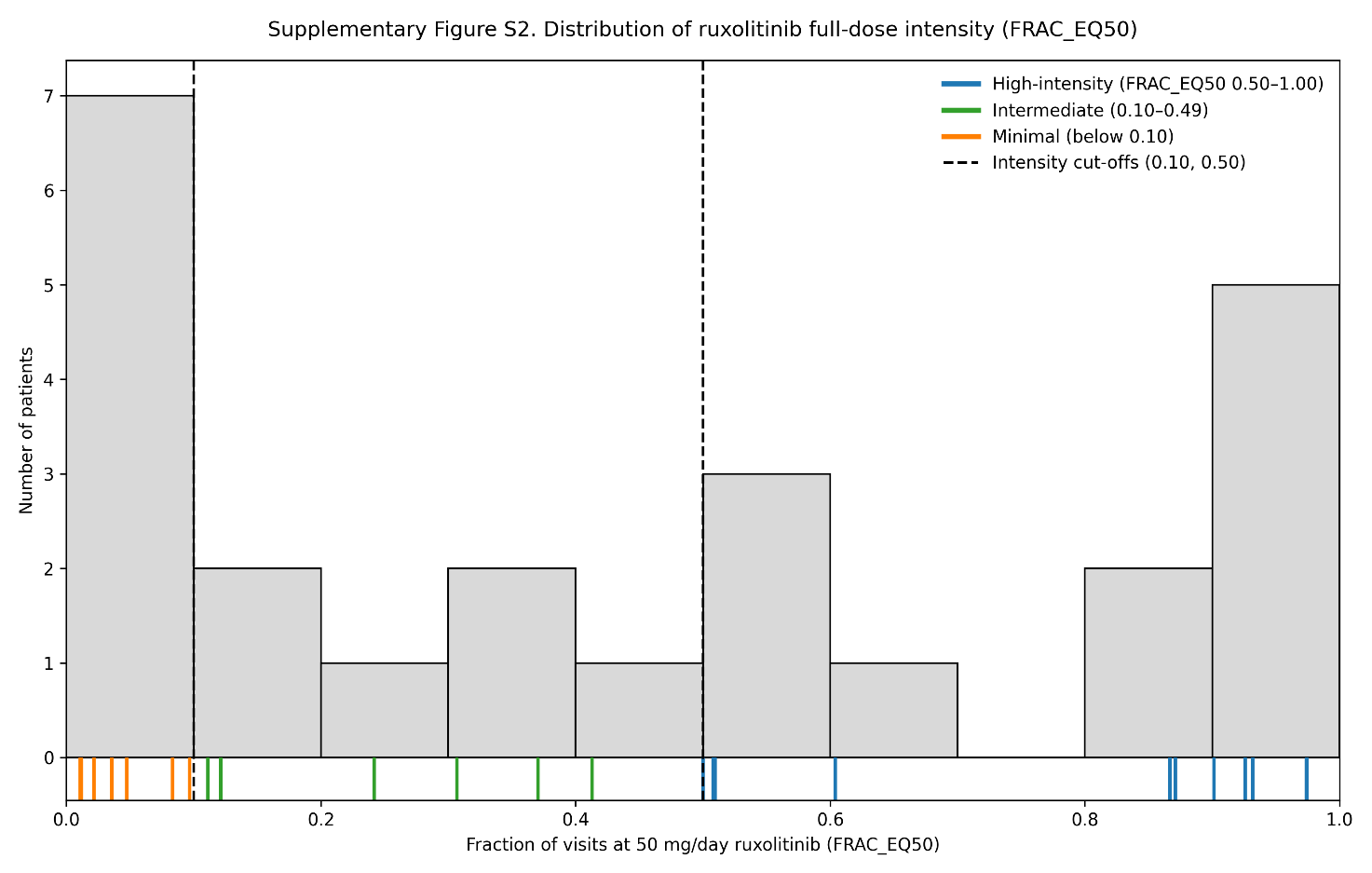


**Supplementary Figure S2.**

**Distribution of ruxolitinib full-dose intensity**

Distribution of full-dose intensity (FRAC_EQ50), defined as the fraction of follow-up visits at exactly 50 mg/day. The histogram summarizes FRAC_EQ50 across patients; colored rug marks indicate individual patients classified as High (FRAC_EQ50 0.50–1.00), Intermediate (0.10–0.49), or Minimal (<0.10). Vertical dashed lines indicate the 0.10 and 0.50 cut-offs used to define the three categories.


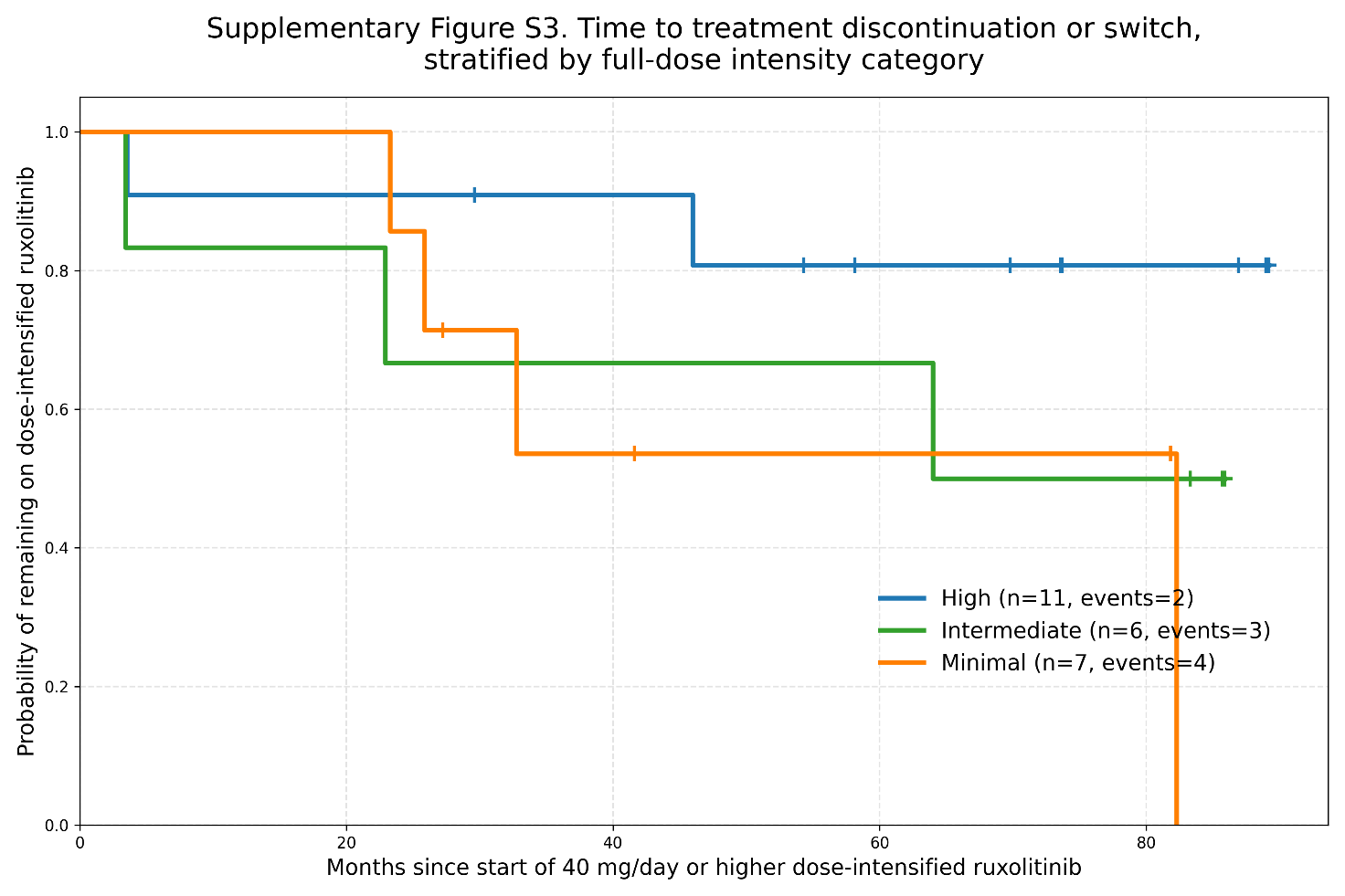


**Supplementary Figure S3.**

**Time to treatment discontinuation or switch**

Kaplan–Meier estimates of time from t0 (first visit at 40 mg/day or higher) to treatment discontinuation or switch to another JAK inhibitor, stratified by full-dose intensity category based on FRAC_EQ50: high (0.50–1.00), intermediate (0.10–0.49), and minimal (below 0.10). Tick marks indicate censored observations at last follow-up while remaining on ruxolitinib. Group sizes (n) and number of events are reported in the legend. Time to treatment discontinuation or switch was estimated using standard Kaplan–Meier methods. Given the retrospective design and the absence of time-dependent modeling of dose exposure, these analyses should be interpreted with caution.
