# Supplementary material for: High-dose ruxolitinib (25 mg twice daily) in myelofibrosis: feasibility, safety, and long-term treatment exposure in a real-world cohort
Source: Ann Hematol. 2026 May 4;105(5):284. doi: 10.1007/s00277-026-07020-1 (PMC13139260; doi:10.1007/s00277-026-07020-1)
Supplement: Supplementary file 2 — Supplementary file2 (DOCX 17 KB) [file 277_2026_7020_MOESM2_ESM.docx]

**SUPPLEMENTARY TABLE 1**

**Supplementary Table 1. Detailed baseline characteristics and treatment-related variables (N = 24)**

| **Variable** | **Value** |
| --- | --- |
| Age, years | **69 (61–76)** |
| Sex (M/F) | **16 / 8** |
| MF subtype |  |
| Primary MF | **14 (58.3%)** |
| Post-PV MF | **7 (29.2%)** |
| Post-ET MF | **3 (12.5%)** |
| Driver mutation |  |
| JAK2 V617F | **19 (79.2%)** |
| CALR | **3 (12.5%)** |
| MPL | **1 (4.2%)** |
| Triple-negative | **1 (4.2%)** |
| DIPSS score (median, IQR) | **4 (3–5)** |
| DIPSS category |  |
| Low | **2 (8.3%)** |
| Intermediate-1 | **7 (29.2%)** |
| Intermediate-2 | **10 (41.7%)** |
| High | **5 (20.8%)** |
| Hemoglobin, g/dL (baseline) | **13.0 (11.4–13.8)** |
| Platelets, ×10⁹/L (baseline) | **474.5 (331.5–772.3)** |
| WBC, ×10⁹/L (baseline) | **9.8 (6.4–14.7)** |
| Baseline spleen length, cm | **14.0 (11.5–17.0)** |
| Baseline spleen available | **22/24 (91.7%)** |
| Prior cytoreductive therapy (any) | **18 (75.0%)** |
| Prior hydroxyurea exposure | **16 (66.7%)** |
| Baseline transfusion dependence | **3 (12.5%)** |
| Baseline ESA use | **9 (37.5%)** |
| Ruxolitinib starting dose, mg/day | **40 (30–50)** |
| Full-dose exposure (25 mg twice daily) | **FRAC_EQ50 values were categorized as high (0.50–1.00), intermediate (0.10–0.49), and minimal (<0.10) dose-intensity exposure.** |
